# Crystal structure and Hirshfeld surface analysis of 2-{[7-acetyl-8-(4-chloro­phen­yl)-4-cyano-6-hy­droxy-1,6-dimethyl-5,6,7,8-tetra­hydro­isoquinolin-3-yl]sulfan­yl}-*N*-(4-chloro­phen­yl)acetamide

**DOI:** 10.1107/S2056989021003674

**Published:** 2021-04-13

**Authors:** Mehmet Akkurt, Islam S. Marae, Joel T. Mague, Shaaban K. Mohamed, Etify A. Bakhite, Safiyyah A. H. Al-Waleedy

**Affiliations:** a Department of Physics, Faculty of Sciences, Erciyes University, 38039 Kayseri, Turkey; b Chemistry Department, Faculty of Science, Assiut University, 71516 Assiut, Egypt; c Department of Chemistry, Tulane University, New Orleans, LA 70118, USA; d Chemistry and Environmental Division, Manchester Metropolitan University, Manchester, M1 5GD, England; e Chemistry Department, Faculty of Science, Minia University, 61519 El-Minia, Egypt; f Department of Chemistry, Faculty of Science, Taiz University, Taiz, Yemen

**Keywords:** crystal structure, tetra­hydro­iso­quinoline, hydrogen bond, twist-boat conformation, Hirshfeld surface analysis

## Abstract

In the title mol­ecule, C_28_H_25_Cl_2_N_3_O_3_S, the heterocyclic portion of the tetra­hydro­iso­quinoline unit is planar while the cyclo­hexene ring adopts a twist-boat conformation. The two 4-chloro­phenyl groups extend away from one side of this unit while the hydroxyl and acetyl groups extend away from the opposite side and form an intra­molecular O—H⋯O hydrogen bond. The crystal packing consists of layers parallel to the *bc* plane. A Hirshfeld surface analysis of the crystal structure indicates that the most important contributions to the crystal packing are from H⋯H (37.3%), Cl⋯H/H⋯Cl (17.6%), O⋯H/H⋯O (11.1%), C⋯H/H⋯C (10.9%) and N⋯H/H⋯N (9.7%) inter­actions.

## Abstract 2

The cyclo­hexene ring of the tetra­hydro­iso­quinoline unit is non-planar. The two 4-chloro­phenyl groups extend away from one side of this unit while the hydroxyl and acetyl groups extend away from the opposite side. An intra­molecular O—H⋯O hydrogen bond fixes the rotational orientation of the acetyl group. In the crystal, N—H⋯O hydrogen bonds form chains of mol­ecules extending along the *c*-axis direction. Inversion-related chains pack to form layers parallel to the *bc* plane.

## Chemical context   

The tetra­hydro­iso­quinoline motif is present in a variety of natural products, including cactus alkaloids (peyoruvic acid; Chrzanowska *et al.*, 1987 ▸) and mammalian alkaloids (salsoline carb­oxy­lic acid; Czarnocki *et al.*, 1992 ▸). Biological tests indicate that tetra­hydro­iso­quinolines can act as bronchodilators (Houston & Rodger, 1974 ▸) and anti­convulsants (Ohkubo *et al.*, 1996 ▸; Thompson *et al.*, 1990 ▸) and they have also shown anti-hypoxic activity (Gill *et al.*, 1991 ▸). Based on these findings and following our inter­est in this area, we herein report the synthesis and crystal structure of the title compound.
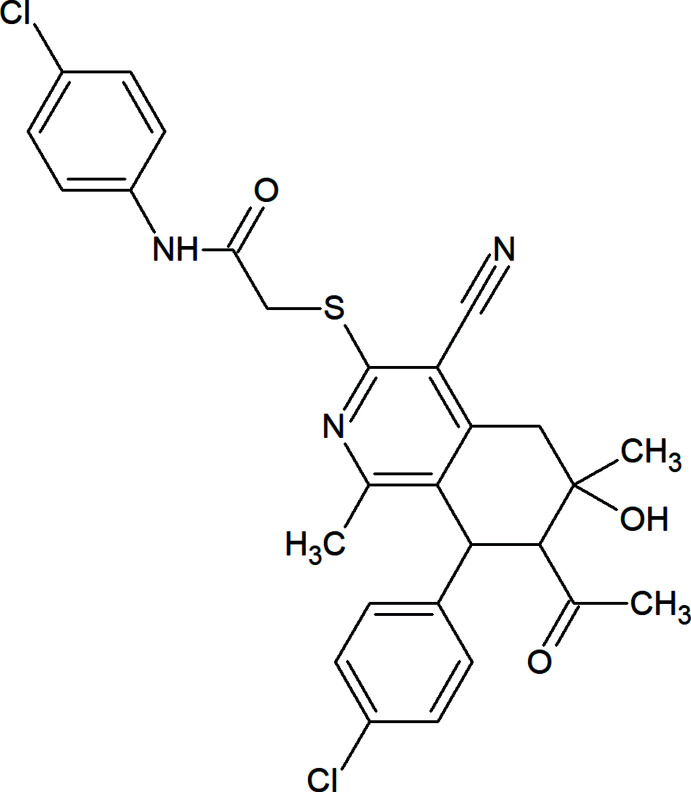


## Structural commentary   

**Figure 1:**
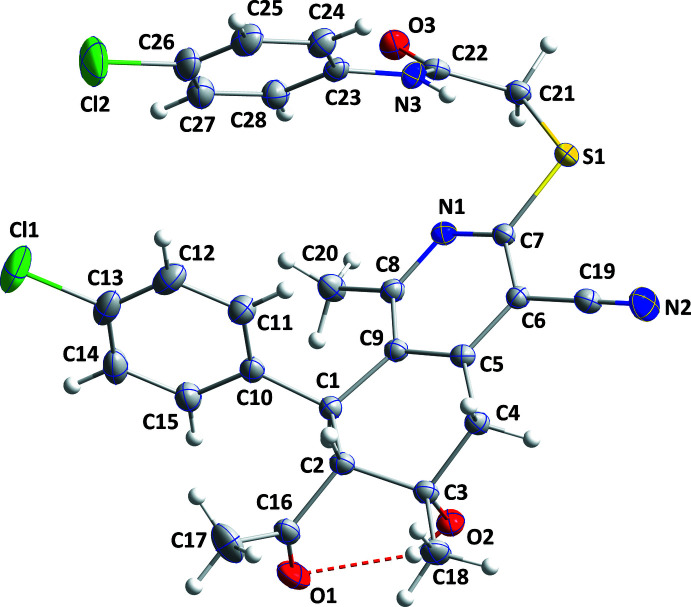
The title mol­ecule with the labeling scheme and 50% probability ellipsoids. The intra­molecular O—H⋯O hydrogen bond is depicted by a dashed line.

The overall conformation of the title mol­ecule, Fig. 1 ▸, resembles that of a chair with the tetra­hydro­iso­quinoline core forming the seat, the hydroxyl and acetyl oxygen atoms forming stubby legs and the 4-chloro­phenyl group and the amide group forming the back. The N1/C5–C9 ring is essentially planar (r.m.s. deviation = 0.041 Å) with the largest deviation of 0.059 (1) Å being for atom C9. A puckering analysis (Cremer & Pople, 1975 ▸) of the C1–C5/C9 ring yielded the following parameters: *Q*_T_ = 0.5230 (13) Å, θ = 54.39 (14)° and φ = 96.94 (17)°. The conformation of this ring approximates a twist-boat conformation. The best planes through the C10–C15 and C23–C28 rings are inclined to the N1/C5–C9 plane by 76.05 (6) and 74.04 (6)°, respectively. The acetyl group on C2 is in an equatorial position while the hydroxyl group on C3 is axial and these are *syn* to one another. The C10–C15 ring attached to C1 is close to equatorial and *anti* with respect to both other substituents (Table 1 ▸, Fig. 1 ▸). The O2—H2*A* hydroxyl group is favorably oriented for forming an intra­molecular hydrogen bond with O1 (Fig. 1 ▸). This was not seen for some related mol­ecules where a stronger intermolecular interaction is favored for these O atoms (Al-Taifi *et al.*, 2021 ▸).

## Supra­molecular features and Hirshfeld surface analysis   

**Figure 2:**
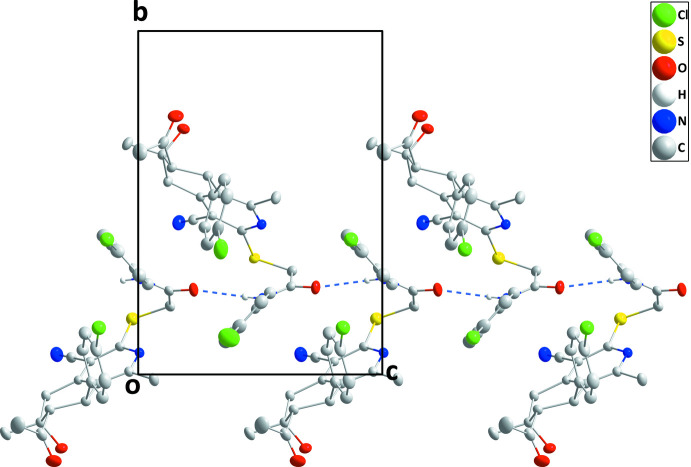
A portion of one chain viewed along the *a*-axis direction with the inter­molecular N—H⋯O hydrogen bonds depicted by dashed lines.

**Figure 3:**
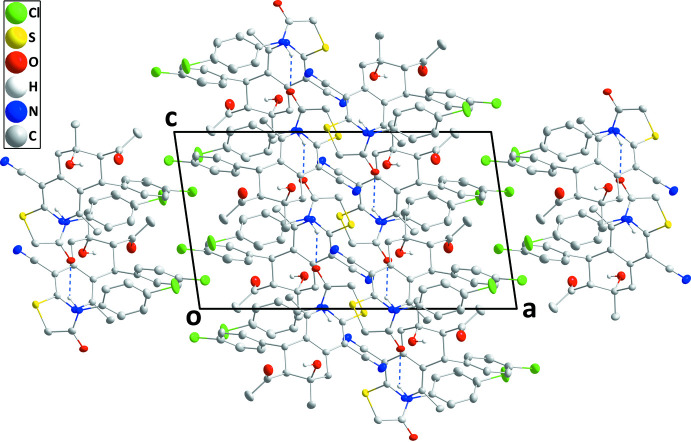
Packing viewed along the *b*-axis direction with N—H⋯O hydrogen bonds depicted by dashed lines.

In the crystal, helical chains extending along the *c*-axis direction are formed by N3—H3⋯O3 hydrogen bonds (Table 1 ▸ and Fig. 2 ▸). Inversion-related chains pack together to form thick layers, which have the chlorine atoms on the outsides (Fig. 3 ▸). In addition, a C22—O3⋯*Cg*1^ii^ inter­action [C22—O3 = 1.3576 (15) Å, O3⋯*Cg*1^ii^ = 3.6287 (11) Å and C22—O3⋯*Cg*1^ii^ = 115.38 (8)°; symmetry code: (ii) *x*, 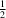 − *y*, 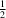 + *z*; where *Cg*1 is the centroid of the N1/C5-C9 ring) are also observed in the crystal structure.

**Figure 4:**
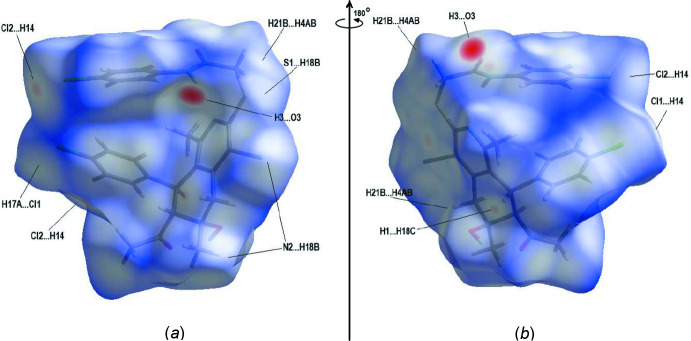
(*a*) Front and (*b*) back sides of the three-dimensional Hirshfeld surface of the title compound plotted over *d*_norm_ in the range −0.3918 to +1.6138 a.u.

The inter­molecular inter­actions in the crystal of the title compound were investigated and visualized by performing a Hirshfeld surface analysis (Spackman & Jayatilaka, 2009 ▸) using *Crystal Explorer 17.5* (Turner *et al.*, 2017 ▸). The Hirshfeld surface plotted over *d*_norm_ in the range −0.3918 to +1.6138 a.u. is shown in Fig. 4 ▸ with red areas indicating distances shorter (in closer contact) and blue those longer (distant contact) than the van der Waals separations. The closest contacts are listed in Table 2 ▸. The O—H⋯O and N—H⋯O hydrogen bonds are clearly shown by the dark-red circles (Tables 1 ▸ and 2 ▸; Fig. 4 ▸).

**Figure 5:**
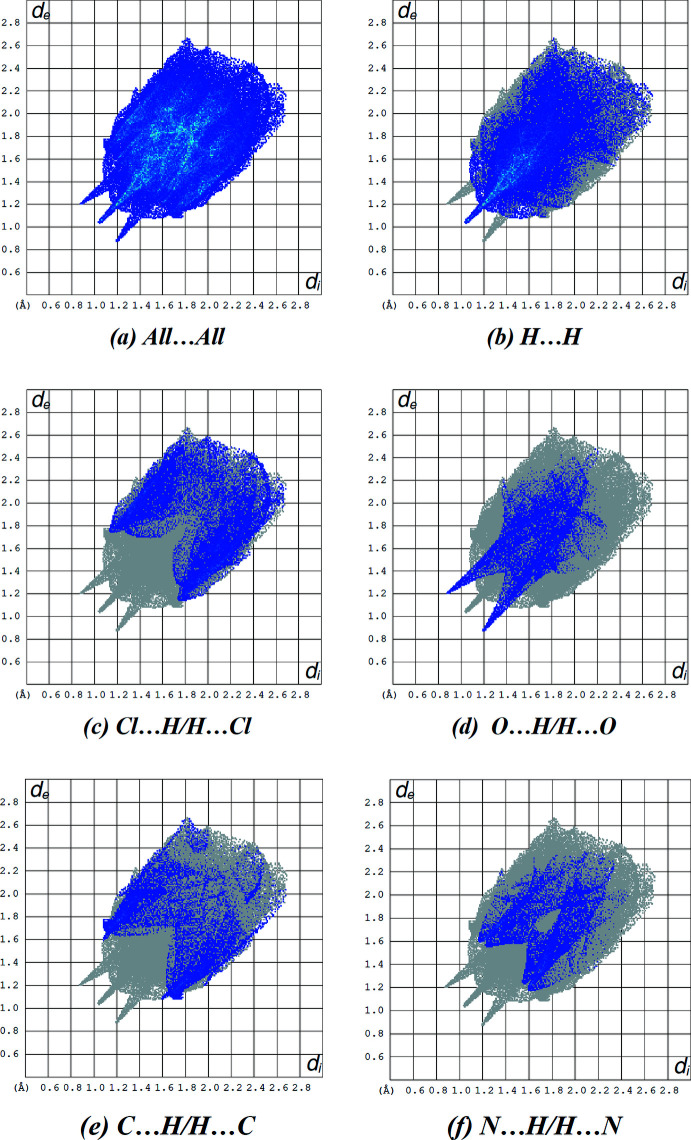
A view of the two-dimensional fingerprint plots for the title compound, showing (*a*) all inter­actions, and delineated into (*b*) H⋯H, (*c*) Cl⋯H/H⋯Cl, (*d*) O⋯H/H⋯O, (*e*) C⋯H/H⋯C and (*f*) N⋯H/H⋯N inter­actions. The *d*_i_ and *d*_e_ values are the closest inter­nal and external distances (in Å) from given points on the Hirshfeld surface.

Fig. 5 ▸ shows the full two-dimensional fingerprint plot (McKinnon *et al.*, 2007 ▸) and those delineated into the major contacts: H⋯H (37.3%; Fig. 5 ▸ *b*), Cl⋯H/H⋯Cl (17.6%; Fig. 5 ▸ *c*), O⋯H/H⋯O (11.1%; Fig. 5 ▸ *d*), C⋯H/H⋯C (10.9%; Fig. 5 ▸ *e*) and N⋯H/H⋯N (9.7%; Fig. 5 ▸ *f*). The other contacts are negligible with individual contributions of less than 2.9% and are given in Table 3 ▸.

## Database survey   

A survey of the Cambridge Structural Database (CSD, version 5.42, November 2020; Groom *et al.*, 2016 ▸) reveals nine comparable tetra­hydro­iso­quinoline derivatives, 7-acetyl-8-(4-chloro­phen­yl)-3-(ethyl­sulfan­yl)-6-hy­droxy-1,6-dimethyl-5,6,7,8-tetra­hydro­iso­quinoline-4-carbo­nitrile (refcode NAQRIJ: Mague *et al.*, 2017 ▸), 2-methyl-1,2,3,4-tetra­hydro­iso­quinoline trihydrate (KUGLIK: Langenohl *et al.*, 2020 ▸), 2′-benzoyl-1′-(4-meth­oxy­phen­yl)-1-methyl-1′,5′,6′,10′b-tetra­hydro-2′*H*-spiro­[indole-3,3′-pyrrolo­[2,1-*a*]isoquinolin]-2(1*H*)-one (DUSVIZ: Selvaraj *et al.*, 2020 ▸), 2-[(7-acetyl-4-cyano-6-hy­droxy-1,6-dimethyl-8-phenyl-5,6,7,8-tetra­hydro­isoquinolin-3-yl)sulfan­yl]-*N*-phenyl­acetamide (AKIVUO: Al-Taifi *et al.*, 2021 ▸), 3-amino-1-oxo-2,6,8-triphenyl-1,2,7,8-tetra­hydro­iso­quinoline-4-carbo­nitrile (ULUTAZ: Naghiyev *et al.*, 2021 ▸), 4-fluoro-3-(4-meth­oxy­phen­yl)-1-oxo-2-phenyl-1,2,3,4-tetra­hy­dro­iso­quinoline-4-carb­oxy­lic acid (CARCOQ: Lehmann *et al.*, 2017 ▸), 2-[3-methyl-4-phenyl-3,4-di­hydro­isoquinolin-2(1*H*)-yl]-1,2-di­phenyl­ethan-1-ol (POPYEB: Ben Ali & Retailleau, 2019 ▸), (1*R*,3*S*)-6,7-dimeth­oxy-3-(meth­oxy­diphenyl­meth­yl)-1-phenyl-1,2,3,4-tetra­hydro­iso­quinoline (ENOCIU: Naicker *et al.*, 2011 ▸) and 1,2,3,4-tetra­hydro­iso­quinoline-2-sulfonamide (NIWPAL: Bouasla *et al.*, 2008 ▸).

In the crystal of NAQRIJ, dimers form through complementary sets of inversion-related O—H⋯O and C—H⋯O hydrogen bonds. These are connected into zigzag chains along the *c*-axis direction by pairwise C—H⋯N inter­actions that also form inversion dimers. In the crystal of KUGLIK, the heterocyclic amines are alternately connected to the hydrogen-bonding system along the *c* axis, which leads to the formation of syndiotactic polymer chains in this direction. The hydrogen-bonding network of the water mol­ecules forms a water plane along the *b* and *c* axes with different ring systems (only counting the oxygen atoms) and graph-set motifs of the hydrogen-bonding network. In the crystal of DUSVIZ, mol­ecules are linked *via* C—H⋯O hydrogen bonds. For the major disorder component, these form *C*(11) chains that propagate parallel to the *a* axis. In the crystal of AKIVUO, a layer structure with the layers parallel to (10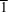) is generated by O—H⋯O and C—H⋯O hydrogen bonds. In the crystal of ULUTAZ, mol­ecules are linked *via* N—H⋯O and C—H⋯N hydrogen bonds, forming a three-dimensional network. Furthermore, the crystal packing is dominated by C—H⋯π bonds with a strong inter­action involving the phenyl H atoms. In the crystal of CARCOQ, mol­ecules are linked by O—H⋯O hydrogen bonds, forming chains propagating along the *a*-axis direction. The chains are linked by C—H⋯F hydrogen bonds, forming layers lying parallel to the *ab* plane. In the crystal of POPYEB, mol­ecules are packed in a herringbone manner parallel to (103) and (10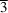) *via* weak C—H⋯O and C—H⋯π(ring) inter­actions. In the crystal structure of ENOCIU, various C—H⋯π and C—H⋯O inter­actions link the mol­ecules. In the crystal of NIWPAL, the mol­ecules are linked by N—H⋯O inter­molecular hydrogen bonds involving the sulfonamide function to form an infinite two-dimensional network parallel to the (001) plane.

## Synthesis and crystallization   

**Figure 6:**
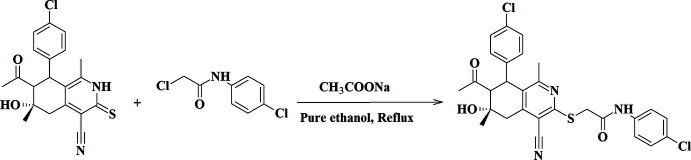
Synthesis scheme for 2-{[7-acetyl-8-(4-chloro­phen­yl)-4-cyano-6-hy­droxy-1,6-dimethyl-5,6,7,8-tetra­hydro­isoquinolin-3-yl]sulfan­yl}-*N*-(4-chloro­phen­yl)acetamide.

The title compound was obtained by refluxing of 7-acetyl-8-(4-chloro­phen­yl)-4-cyano-1,6-dimethyl-6-hy­droxy-5,6,7,8-tetra­hydro­iso­quinoline-3(2*H*)-thione, (0.77 g, 2 mmol) with *N*-(4-chloro­phen­yl)-2-chloro­acetamide (0.40 g, 2 mmol) and (0.98 g, 12 mmol) of anhydrous sodium acetate in pure ethanol (30 ml) for 1 h as shown in Fig. 6 ▸. The product that formed during cooling was collected and recrystallized from ethanol to give good quality crystals suitable for X-ray diffraction. Yield: 1.00 g, 91%; m.p. 491–493 K.

IR: 3522 cm^−1^ (O—H), 3277 cm^−1^ (N—H), 2991, 2920 cm^−1^ (C—H, aliphatic), 2217 cm^−1^ (C≡N), 1694 (C=O, acet­yl), 1666 cm^−1^ (C=O, amide). ^1^H NMR (400 MHz, DMSO-*d_6_*): δ 10.95 (*s*, 1H, NH); 8.17–8.24 (*m*, 2H, Ar-H); 7.79–7.81 (*d*, 2H, Ar-H); 7.26–7.32 (*m*, 2H, Ar-H); 7.03–7.05 (*d*, 2H, Ar-H); 4.88 (*s*, 1H, OH); 4.53–4.55 (*d*, 1H, CH at C-8); 4.19–4.20 (*dd*, 2H, SCH_2_); 3.24–3.29 (*d*, 1H, CH at C-5); 2.87–2.90 (*m*, 2H: CH at C-5 and CH at C-7); 2.13 (*s*, 3H, COCH_3_); 1.86 (*s*, 3H, CH_3_ attached to pyridine ring); 1.27 (*s*, 3H, CH_3_). Analysis calculated for C_28_H_25_Cl_2_N_3_O_3_S (554.47): C 60.65%, H 4.54%, N 7.58%, S 5.78%. Found: C 60.34%, H 4.57%, N 7.68%, S 5.97%.

## Refinement   

Crystal data, data collection and structure refinement details are summarized in Table 4 ▸. All C-bound H atoms were placed in geometrically idealized positions (C—H = 0.95–1.00 Å) while those attached to O and to N were placed in locations derived from a difference map, refined for a few cycles to ensure that reasonable displacement parameters could be achieved, and then their coordinates were adjusted to give O—H = 0.87 and N—H = 0.91 Å. All H atoms were included as riding contributions with isotropic displacement parameters 1.2–1.5 times those of the parent atoms.

## Supplementary Material

Crystal structure: contains datablock(s) I, global. DOI: 10.1107/S2056989021003674/vm2246sup1.cif

Structure factors: contains datablock(s) I. DOI: 10.1107/S2056989021003674/vm2246Isup2.hkl

Supporting information file. DOI: 10.1107/S2056989021003674/vm2246Isup3.cml

CCDC reference: 2075592

Additional supporting information:  crystallographic information; 3D view; checkCIF report

## Figures and Tables

**Table 1:** Hydrogen-bond geometry (Å, °)

*D*—H⋯*A*	*D*—H	H⋯*A*	*D*⋯*A*	*D*—H⋯*A*
O2—H2*A*⋯O1	0.87	2.14	2.8746 (14)	142
N3—H3⋯O3^i^	0.91	2.17	2.9362 (13)	141

Symmetry code: (i) x, -y+{\script{1\over 2}}, z-{\script{1\over 2}}.

**Table 2:** Summary of short inter­atomic contacts (Å) in the title compound

Contact	Distance	Symmetry operation
Cl2⋯H14	3.06	-*x*, −{1\over 2} + *y*, {1\over 2} − *z*;
H14⋯Cl1	2.98	-*x*, 1 − *y*, 1 − *z*;
H17*A*⋯Cl1	3.02	-*x*, 1 − *y*, −*z*;
S1⋯H18*B*	3.17	1 − *x*, −{1\over 2} + *y*, {1\over 2} − *z*;
H21*B*⋯H4*AB*	2.51	1 − *x*, 1 − *y*, 1 − *z*;
H3⋯O3	2.17	*x*, {1\over 2} − *y*, −{1\over 2} + *z*;
H1⋯H18*C*	2.26	*x*, {3\over 2} − *y*, {1\over 2} + *z*;
H18*B*⋯N2	2.86	1 − *x*, 1 − *y*, −*z*.

**Table 3:** Percentage contributions of inter­atomic contacts to the Hirshfeld surface for the title compound

Contact	Percentage contribution
H⋯H	37.3
Cl⋯H/H⋯Cl	17.6
O⋯H/H⋯O	11.1
C⋯H/H⋯C	10.9
N⋯H/H⋯N	9.7
S⋯H/H⋯S	2.9
Cl⋯C/C⋯Cl	1.7
O⋯C/C⋯O	1.6
S⋯C/C⋯S	1.6
Cl⋯O/O⋯Cl	1.6
C⋯C	1.3
N⋯C/C⋯N	1.1
S⋯O/O⋯S	0.8
S⋯N/N⋯S	0.4
N⋯N	0.2
Cl⋯Cl	0.2
O⋯N/N⋯O	0.1

**Table 4:** Experimental details

Crystal data	
Chemical formula	C_28_H_25_Cl_2_N_3_O_3_S
*M*_r_	554.47
Crystal system, space group	Monoclinic, *P*2_1_/*c*
Temperature (K)	150
*a*, *b*, *c* (Å)	18.2076 (8), 14.2859 (6), 10.2713 (5)
β (°)	98.245 (1)
*V* (Å^3^)	2644.1 (2)
*Z*	4
Radiation type	Mo *K*α
μ (mm^−1^)	0.36
Crystal size (mm)	0.29 × 0.21 × 0.17

Data collection	
Diffractometer	Bruker *SMART* *APEX* CCD
Absorption correction	Multi-scan (*SADABS*; Krause *et al.*, 2015 ▸)
*T*_min_, *T*_max_	0.85, 0.94
No. of measured, independent and observed [*I* > 2σ(*I*)] reflections	50860, 7143, 5685
*R*_int_	0.037
(sin θ/λ)_max_ (Å^−1^)	0.689

Refinement	
*R*[*F* ^2^ > 2σ(*F* ^2^)], *wR*(*F* ^2^), *S*	0.041, 0.115, 1.07
No. of reflections	7143
No. of parameters	337
H-atom treatment	H-atom parameters constrained
Δρ_max_, Δρ_min_ (e Å^−3^)	0.55, −0.22

Computer programs: *APEX3* and *SAINT* (Bruker, 2016 ▸), *SHELXT* (Sheldrick, 2015*a* ▸), *SHELXL2018/1* (Sheldrick, 2015*b* ▸), *DIAMOND* (Brandenburg & Putz, 2012 ▸) and *SHELXTL* (Sheldrick, 2008 ▸).

## supplementary crystallographic information

### Crystal data

**Table:**

C_28_H_25_Cl_2_N_3_O_3_S	*F*(000) = 1152
*M**_r_* = 554.47	*D*_x_ = 1.393 Mg m^−^^3^
Monoclinic, *P*2_1_/*c*	Mo *K*α radiation, λ = 0.71073 Å
*a* = 18.2076 (8) Å	Cell parameters from 9990 reflections
*b* = 14.2859 (6) Å	θ = 2.5–29.3°
*c* = 10.2713 (5) Å	µ = 0.36 mm^−^^1^
β = 98.245 (1)°	*T* = 150 K
*V* = 2644.1 (2) Å^3^	Column, colourless
*Z* = 4	0.29 × 0.21 × 0.17 mm

### Data collection

**Table:**

Bruker SMART APEX CCD diffractometer	7143 independent reflections
Radiation source: fine-focus sealed tube	5685 reflections with *I* > 2σ(*I*)
Graphite monochromator	*R*_int_ = 0.037
Detector resolution: 8.3333 pixels mm^-1^	θ_max_ = 29.3°, θ_min_ = 1.8°
φ and ω scans	*h* = −24→25
Absorption correction: multi-scan (*SADABS*; Krause *et al.*, 2015)	*k* = −19→19
*T*_min_ = 0.85, *T*_max_ = 0.94	*l* = −14→14
50860 measured reflections	

### Refinement

**Table:**

Refinement on *F*^2^	Primary atom site location: dual
Least-squares matrix: full	Secondary atom site location: difference Fourier map
*R*[*F*^2^ > 2σ(*F*^2^)] = 0.041	Hydrogen site location: mixed
*wR*(*F*^2^) = 0.115	H-atom parameters constrained
*S* = 1.07	*w* = 1/[σ^2^(*F*_o_^2^) + (0.0726*P*)^2^ + 0.1632*P*] where *P* = (*F*_o_^2^ + 2*F*_c_^2^)/3
7143 reflections	(Δ/σ)_max_ = 0.001
337 parameters	Δρ_max_ = 0.55 e Å^−^^3^
0 restraints	Δρ_min_ = −0.22 e Å^−^^3^

### Special details

**Table:**

Experimental. The diffraction data were obtained from 3 sets of 400 frames, each of width 0.5° in ω, colllected at φ = 0.00, 90.00 and 180.00° and 2 sets of 800 frames, each of width 0.45° in φ, collected at ω = –30.00 and 210.00°. The scan time was 20 sec/frame.
Geometry. All esds (except the esd in the dihedral angle between two l.s. planes) are estimated using the full covariance matrix. The cell esds are taken into account individually in the estimation of esds in distances, angles and torsion angles; correlations between esds in cell parameters are only used when they are defined by crystal symmetry. An approximate (isotropic) treatment of cell esds is used for estimating esds involving l.s. planes.
Refinement. Refinement of F^2^ against ALL reflections. The weighted R-factor wR and goodness of fit S are based on F^2^, conventional R-factors R are based on F, with F set to zero for negative F^2^. The threshold expression of F^2^ > 2sigma(F^2^) is used only for calculating R-factors(gt) etc. and is not relevant to the choice of reflections for refinement. R-factors based on F^2^ are statistically about twice as large as those based on F, and R- factors based on ALL data will be even larger. H-atoms attached to carbon were placed in calculated positions (C—H = 0.95 - 1.00 Å) while those attached to nitrogen and to oxygen were placed in locations derived from a difference map and their coordinates adjusted to give N—H = 0.91 and O—H = 0.87 %A. All were included as riding contributions with isotropic displacement parameters 1.2 - 1.5 times those of the attached atoms.

### Fractional atomic coordinates and isotropic or equivalent isotropic displacement parameters (Å^2^)

**Table:**

	*x*	*y*	*z*	*U*_iso_*/*U*_eq_	
Cl1	−0.02990 (2)	0.36422 (4)	0.33858 (4)	0.04981 (14)	
Cl2	0.06724 (2)	0.10721 (4)	0.37194 (6)	0.06092 (16)	
S1	0.51601 (2)	0.33832 (2)	0.47637 (3)	0.02002 (9)	
O1	0.18797 (6)	0.75204 (7)	0.15363 (11)	0.0362 (2)	
O2	0.34473 (5)	0.71519 (6)	0.18526 (9)	0.0251 (2)	
H2A	0.306643	0.752176	0.183434	0.038*	
O3	0.38487 (5)	0.25324 (7)	0.72696 (9)	0.0266 (2)	
N3	0.38125 (6)	0.22031 (7)	0.50946 (10)	0.0202 (2)	
H3	0.405602	0.226236	0.438511	0.024*	
N1	0.39445 (6)	0.43662 (7)	0.50524 (10)	0.0195 (2)	
N2	0.54848 (7)	0.44181 (10)	0.16630 (12)	0.0346 (3)	
C1	0.25066 (6)	0.57818 (8)	0.28505 (12)	0.0182 (2)	
H1	0.250642	0.638103	0.335370	0.022*	
C2	0.24356 (7)	0.60275 (8)	0.13694 (12)	0.0192 (2)	
H2	0.229573	0.544713	0.084806	0.023*	
C3	0.31715 (7)	0.64019 (9)	0.09934 (12)	0.0193 (2)	
C4	0.37436 (7)	0.56220 (9)	0.12178 (12)	0.0197 (2)	
H4A	0.362155	0.513961	0.052868	0.024*	
H4AB	0.423739	0.588126	0.112304	0.024*	
C5	0.37853 (6)	0.51651 (8)	0.25450 (11)	0.0169 (2)	
C6	0.44083 (6)	0.46236 (8)	0.30248 (12)	0.0175 (2)	
C7	0.44414 (6)	0.41916 (8)	0.42543 (12)	0.0178 (2)	
C8	0.33611 (6)	0.49192 (8)	0.46270 (12)	0.0189 (2)	
C9	0.32273 (6)	0.52698 (8)	0.33326 (11)	0.0175 (2)	
C10	0.18187 (7)	0.52296 (9)	0.30652 (12)	0.0210 (2)	
C11	0.17522 (8)	0.42867 (10)	0.27233 (14)	0.0270 (3)	
H11	0.215512	0.397364	0.241791	0.032*	
C12	0.11017 (8)	0.37991 (11)	0.28247 (15)	0.0335 (3)	
H12	0.105907	0.315681	0.258635	0.040*	
C13	0.05205 (8)	0.42535 (12)	0.32726 (14)	0.0331 (3)	
C14	0.05739 (7)	0.51863 (12)	0.36358 (14)	0.0329 (3)	
H14	0.017144	0.549228	0.395275	0.040*	
C15	0.12252 (7)	0.56685 (10)	0.35299 (14)	0.0273 (3)	
H15	0.126618	0.630890	0.377902	0.033*	
C16	0.18163 (7)	0.67507 (10)	0.10366 (13)	0.0249 (3)	
C17	0.11592 (9)	0.64770 (13)	0.00686 (17)	0.0427 (4)	
H17A	0.131638	0.636429	−0.079127	0.064*	
H17B	0.079144	0.698219	−0.000773	0.064*	
H17C	0.093878	0.590491	0.036992	0.064*	
C18	0.30814 (8)	0.67229 (10)	−0.04373 (13)	0.0281 (3)	
H18A	0.288390	0.620672	−0.101182	0.042*	
H18B	0.356492	0.691427	−0.066072	0.042*	
H18C	0.273755	0.725379	−0.055836	0.042*	
C19	0.50033 (7)	0.45056 (9)	0.22598 (12)	0.0216 (3)	
C20	0.28761 (7)	0.51467 (10)	0.56440 (12)	0.0259 (3)	
H20A	0.267277	0.577862	0.548872	0.039*	
H20B	0.316951	0.511668	0.652083	0.039*	
H20C	0.246849	0.469388	0.558811	0.039*	
C21	0.48904 (7)	0.30133 (9)	0.63011 (12)	0.0211 (2)	
H21A	0.526562	0.256096	0.671259	0.025*	
H21B	0.491187	0.356564	0.688747	0.025*	
C22	0.41320 (7)	0.25678 (8)	0.62599 (12)	0.0200 (2)	
C23	0.30606 (7)	0.19224 (9)	0.48223 (12)	0.0208 (2)	
C24	0.28725 (8)	0.12164 (10)	0.38993 (13)	0.0261 (3)	
H24	0.324845	0.091634	0.349871	0.031*	
C25	0.21356 (8)	0.09505 (10)	0.35639 (15)	0.0323 (3)	
H25	0.200533	0.046510	0.294129	0.039*	
C26	0.15947 (8)	0.13995 (11)	0.41455 (16)	0.0338 (3)	
C27	0.17712 (8)	0.21117 (11)	0.50392 (15)	0.0321 (3)	
H27	0.139056	0.242487	0.541132	0.039*	
C28	0.25087 (7)	0.23702 (10)	0.53944 (13)	0.0263 (3)	
H28	0.263523	0.285112	0.602558	0.032*	

### Atomic displacement parameters (Å^2^)

**Table:**

	*U*^11^	*U*^22^	*U*^33^	*U*^12^	*U*^13^	*U*^23^
Cl1	0.0360 (2)	0.0749 (3)	0.0390 (2)	−0.0295 (2)	0.00696 (17)	0.0015 (2)
Cl2	0.0240 (2)	0.0690 (3)	0.0864 (4)	−0.01335 (19)	−0.0033 (2)	−0.0080 (3)
S1	0.01734 (15)	0.01972 (16)	0.02272 (16)	0.00206 (10)	0.00195 (11)	0.00332 (11)
O1	0.0341 (6)	0.0269 (5)	0.0470 (6)	0.0108 (4)	0.0042 (5)	0.0020 (5)
O2	0.0255 (5)	0.0200 (4)	0.0296 (5)	−0.0013 (3)	0.0039 (4)	−0.0026 (4)
O3	0.0287 (5)	0.0354 (5)	0.0161 (4)	0.0012 (4)	0.0043 (4)	0.0027 (4)
N3	0.0210 (5)	0.0238 (5)	0.0162 (5)	−0.0028 (4)	0.0042 (4)	−0.0008 (4)
N1	0.0211 (5)	0.0195 (5)	0.0177 (5)	0.0002 (4)	0.0022 (4)	0.0015 (4)
N2	0.0323 (7)	0.0426 (8)	0.0314 (7)	0.0089 (5)	0.0125 (5)	0.0027 (5)
C1	0.0173 (5)	0.0184 (6)	0.0191 (6)	0.0012 (4)	0.0029 (4)	0.0008 (4)
C2	0.0197 (6)	0.0187 (6)	0.0184 (6)	0.0017 (4)	0.0006 (4)	0.0021 (4)
C3	0.0207 (6)	0.0193 (6)	0.0179 (6)	0.0017 (4)	0.0026 (4)	0.0019 (4)
C4	0.0209 (6)	0.0221 (6)	0.0164 (5)	0.0033 (4)	0.0043 (4)	0.0022 (4)
C5	0.0193 (5)	0.0158 (5)	0.0154 (5)	0.0000 (4)	0.0016 (4)	−0.0003 (4)
C6	0.0184 (5)	0.0163 (5)	0.0182 (5)	−0.0002 (4)	0.0040 (4)	−0.0008 (4)
C7	0.0171 (5)	0.0165 (5)	0.0193 (6)	0.0004 (4)	0.0005 (4)	0.0010 (4)
C8	0.0199 (5)	0.0193 (6)	0.0178 (5)	−0.0005 (4)	0.0031 (4)	0.0012 (4)
C9	0.0180 (5)	0.0168 (5)	0.0173 (5)	−0.0001 (4)	0.0015 (4)	0.0002 (4)
C10	0.0187 (6)	0.0252 (6)	0.0190 (6)	0.0004 (5)	0.0022 (4)	0.0031 (5)
C11	0.0275 (7)	0.0259 (7)	0.0281 (7)	−0.0024 (5)	0.0057 (5)	−0.0005 (5)
C12	0.0367 (8)	0.0324 (8)	0.0309 (7)	−0.0116 (6)	0.0034 (6)	0.0013 (6)
C13	0.0264 (7)	0.0466 (9)	0.0256 (7)	−0.0129 (6)	0.0018 (5)	0.0062 (6)
C14	0.0204 (6)	0.0488 (9)	0.0305 (7)	0.0008 (6)	0.0068 (5)	0.0044 (6)
C15	0.0228 (6)	0.0304 (7)	0.0292 (7)	0.0026 (5)	0.0058 (5)	0.0015 (5)
C16	0.0217 (6)	0.0304 (7)	0.0229 (6)	0.0051 (5)	0.0041 (5)	0.0082 (5)
C17	0.0278 (8)	0.0559 (11)	0.0405 (9)	0.0102 (7)	−0.0091 (7)	−0.0009 (7)
C18	0.0299 (7)	0.0321 (7)	0.0227 (6)	0.0062 (5)	0.0058 (5)	0.0106 (5)
C19	0.0241 (6)	0.0207 (6)	0.0199 (6)	0.0038 (5)	0.0026 (5)	0.0014 (5)
C20	0.0270 (6)	0.0322 (7)	0.0195 (6)	0.0072 (5)	0.0068 (5)	0.0040 (5)
C21	0.0219 (6)	0.0207 (6)	0.0195 (6)	0.0004 (4)	−0.0012 (5)	0.0025 (5)
C22	0.0234 (6)	0.0178 (6)	0.0181 (6)	0.0031 (4)	0.0010 (4)	0.0029 (4)
C23	0.0222 (6)	0.0218 (6)	0.0182 (6)	−0.0023 (5)	0.0023 (4)	0.0043 (5)
C24	0.0278 (7)	0.0265 (7)	0.0238 (6)	−0.0036 (5)	0.0025 (5)	−0.0012 (5)
C25	0.0324 (7)	0.0308 (7)	0.0318 (7)	−0.0082 (6)	−0.0014 (6)	−0.0025 (6)
C26	0.0231 (7)	0.0382 (8)	0.0384 (8)	−0.0073 (6)	−0.0016 (6)	0.0047 (6)
C27	0.0244 (7)	0.0371 (8)	0.0356 (8)	0.0010 (6)	0.0067 (6)	0.0032 (6)
C28	0.0261 (7)	0.0267 (7)	0.0263 (6)	0.0001 (5)	0.0045 (5)	−0.0001 (5)

### Geometric parameters (Å, º)

**Table:**

Cl1—C13	1.7471 (14)	C10—C11	1.3929 (18)
Cl2—C26	1.7376 (14)	C11—C12	1.3907 (19)
S1—C7	1.7674 (12)	C11—H11	0.9500
S1—C21	1.7989 (13)	C12—C13	1.376 (2)
O1—C16	1.2120 (18)	C12—H12	0.9500
O2—C3	1.4325 (15)	C13—C14	1.383 (2)
O2—H2A	0.8699	C14—C15	1.3891 (19)
O3—C22	1.2238 (15)	C14—H14	0.9500
N3—C22	1.3576 (15)	C15—H15	0.9500
N3—C23	1.4154 (15)	C16—C17	1.494 (2)
N3—H3	0.9096	C17—H17A	0.9800
N1—C7	1.3288 (16)	C17—H17B	0.9800
N1—C8	1.3454 (15)	C17—H17C	0.9800
N2—C19	1.1464 (17)	C18—H18A	0.9800
C1—C9	1.5219 (16)	C18—H18B	0.9800
C1—C10	1.5228 (16)	C18—H18C	0.9800
C1—C2	1.5485 (17)	C20—H20A	0.9800
C1—H1	1.0000	C20—H20B	0.9800
C2—C16	1.5313 (17)	C20—H20C	0.9800
C2—C3	1.5424 (17)	C21—C22	1.5155 (17)
C2—H2	1.0000	C21—H21A	0.9900
C3—C4	1.5201 (16)	C21—H21B	0.9900
C3—C18	1.5256 (17)	C23—C28	1.3906 (19)
C4—C5	1.5034 (16)	C23—C24	1.3929 (18)
C4—H4A	0.9900	C24—C25	1.3894 (19)
C4—H4AB	0.9900	C24—H24	0.9500
C5—C9	1.3945 (17)	C25—C26	1.381 (2)
C5—C6	1.4027 (16)	C25—H25	0.9500
C6—C7	1.3991 (16)	C26—C27	1.377 (2)
C6—C19	1.4365 (17)	C27—C28	1.3901 (19)
C8—C9	1.4089 (16)	C27—H27	0.9500
C8—C20	1.4971 (18)	C28—H28	0.9500
C10—C15	1.3917 (18)		
			
C7—S1—C21	99.68 (6)	C14—C13—Cl1	119.42 (12)
C3—O2—H2A	103.5	C13—C14—C15	118.97 (13)
C22—N3—C23	124.26 (11)	C13—C14—H14	120.5
C22—N3—H3	118.3	C15—C14—H14	120.5
C23—N3—H3	115.9	C14—C15—C10	121.13 (13)
C7—N1—C8	119.05 (10)	C14—C15—H15	119.4
C9—C1—C10	113.12 (10)	C10—C15—H15	119.4
C9—C1—C2	112.04 (10)	O1—C16—C17	122.54 (13)
C10—C1—C2	107.77 (9)	O1—C16—C2	119.68 (12)
C9—C1—H1	107.9	C17—C16—C2	117.76 (12)
C10—C1—H1	107.9	C16—C17—H17A	109.5
C2—C1—H1	107.9	C16—C17—H17B	109.5
C16—C2—C3	110.39 (10)	H17A—C17—H17B	109.5
C16—C2—C1	109.11 (10)	C16—C17—H17C	109.5
C3—C2—C1	111.81 (10)	H17A—C17—H17C	109.5
C16—C2—H2	108.5	H17B—C17—H17C	109.5
C3—C2—H2	108.5	C3—C18—H18A	109.5
C1—C2—H2	108.5	C3—C18—H18B	109.5
O2—C3—C4	106.43 (9)	H18A—C18—H18B	109.5
O2—C3—C18	110.39 (10)	C3—C18—H18C	109.5
C4—C3—C18	110.06 (10)	H18A—C18—H18C	109.5
O2—C3—C2	110.21 (10)	H18B—C18—H18C	109.5
C4—C3—C2	108.00 (10)	N2—C19—C6	179.01 (15)
C18—C3—C2	111.60 (10)	C8—C20—H20A	109.5
C5—C4—C3	113.58 (10)	C8—C20—H20B	109.5
C5—C4—H4A	108.8	H20A—C20—H20B	109.5
C3—C4—H4A	108.8	C8—C20—H20C	109.5
C5—C4—H4AB	108.8	H20A—C20—H20C	109.5
C3—C4—H4AB	108.8	H20B—C20—H20C	109.5
H4A—C4—H4AB	107.7	C22—C21—S1	117.46 (8)
C9—C5—C6	118.36 (10)	C22—C21—H21A	107.9
C9—C5—C4	122.22 (10)	S1—C21—H21A	107.9
C6—C5—C4	119.41 (10)	C22—C21—H21B	107.9
C7—C6—C5	119.23 (11)	S1—C21—H21B	107.9
C7—C6—C19	120.24 (10)	H21A—C21—H21B	107.2
C5—C6—C19	120.52 (11)	O3—C22—N3	123.49 (12)
N1—C7—C6	121.94 (11)	O3—C22—C21	119.13 (11)
N1—C7—S1	118.73 (9)	N3—C22—C21	117.35 (11)
C6—C7—S1	119.32 (9)	C28—C23—C24	119.91 (12)
N1—C8—C9	122.47 (11)	C28—C23—N3	121.55 (11)
N1—C8—C20	114.72 (10)	C24—C23—N3	118.43 (12)
C9—C8—C20	122.81 (11)	C25—C24—C23	120.08 (13)
C5—C9—C8	117.88 (10)	C25—C24—H24	120.0
C5—C9—C1	121.92 (10)	C23—C24—H24	120.0
C8—C9—C1	120.15 (11)	C26—C25—C24	119.27 (13)
C15—C10—C11	118.59 (12)	C26—C25—H25	120.4
C15—C10—C1	120.76 (12)	C24—C25—H25	120.4
C11—C10—C1	120.53 (11)	C27—C26—C25	121.25 (13)
C12—C11—C10	120.64 (14)	C27—C26—Cl2	119.44 (12)
C12—C11—H11	119.7	C25—C26—Cl2	119.30 (12)
C10—C11—H11	119.7	C26—C27—C28	119.71 (14)
C13—C12—C11	119.54 (14)	C26—C27—H27	120.1
C13—C12—H12	120.2	C28—C27—H27	120.1
C11—C12—H12	120.2	C27—C28—C23	119.75 (13)
C12—C13—C14	121.12 (13)	C27—C28—H28	120.1
C12—C13—Cl1	119.45 (12)	C23—C28—H28	120.1
			
C9—C1—C2—C16	−165.26 (10)	C2—C1—C9—C5	8.11 (15)
C10—C1—C2—C16	69.67 (12)	C10—C1—C9—C8	−52.56 (15)
C9—C1—C2—C3	−42.86 (13)	C2—C1—C9—C8	−174.62 (11)
C10—C1—C2—C3	−167.93 (10)	C9—C1—C10—C15	137.21 (12)
C16—C2—C3—O2	69.67 (12)	C2—C1—C10—C15	−98.37 (13)
C1—C2—C3—O2	−51.99 (13)	C9—C1—C10—C11	−46.77 (16)
C16—C2—C3—C4	−174.46 (10)	C2—C1—C10—C11	77.64 (14)
C1—C2—C3—C4	63.88 (12)	C15—C10—C11—C12	1.0 (2)
C16—C2—C3—C18	−53.36 (14)	C1—C10—C11—C12	−175.08 (12)
C1—C2—C3—C18	−175.02 (10)	C10—C11—C12—C13	−0.3 (2)
O2—C3—C4—C5	69.02 (12)	C11—C12—C13—C14	−0.6 (2)
C18—C3—C4—C5	−171.35 (11)	C11—C12—C13—Cl1	179.46 (11)
C2—C3—C4—C5	−49.30 (13)	C12—C13—C14—C15	0.7 (2)
C3—C4—C5—C9	16.26 (16)	Cl1—C13—C14—C15	−179.38 (11)
C3—C4—C5—C6	−162.89 (11)	C13—C14—C15—C10	0.1 (2)
C9—C5—C6—C7	1.48 (17)	C11—C10—C15—C14	−0.9 (2)
C4—C5—C6—C7	−179.33 (11)	C1—C10—C15—C14	175.16 (12)
C9—C5—C6—C19	−179.09 (11)	C3—C2—C16—O1	−59.41 (16)
C4—C5—C6—C19	0.10 (17)	C1—C2—C16—O1	63.84 (15)
C8—N1—C7—C6	5.13 (17)	C3—C2—C16—C17	119.10 (13)
C8—N1—C7—S1	−174.45 (9)	C1—C2—C16—C17	−117.65 (14)
C5—C6—C7—N1	−8.16 (18)	C7—S1—C21—C22	59.47 (10)
C19—C6—C7—N1	172.41 (11)	C23—N3—C22—O3	14.1 (2)
C5—C6—C7—S1	171.42 (9)	C23—N3—C22—C21	−167.77 (11)
C19—C6—C7—S1	−8.01 (16)	S1—C21—C22—O3	−160.99 (10)
C21—S1—C7—N1	3.28 (11)	S1—C21—C22—N3	20.84 (15)
C21—S1—C7—C6	−176.31 (10)	C22—N3—C23—C28	31.66 (18)
C7—N1—C8—C9	4.53 (18)	C22—N3—C23—C24	−152.12 (12)
C7—N1—C8—C20	−174.30 (11)	C28—C23—C24—C25	−0.9 (2)
C6—C5—C9—C8	7.41 (16)	N3—C23—C24—C25	−177.19 (12)
C4—C5—C9—C8	−171.75 (11)	C23—C24—C25—C26	0.6 (2)
C6—C5—C9—C1	−175.26 (11)	C24—C25—C26—C27	0.7 (2)
C4—C5—C9—C1	5.58 (17)	C24—C25—C26—Cl2	179.63 (11)
N1—C8—C9—C5	−10.85 (17)	C25—C26—C27—C28	−1.8 (2)
C20—C8—C9—C5	167.89 (11)	Cl2—C26—C27—C28	179.33 (11)
N1—C8—C9—C1	171.78 (11)	C26—C27—C28—C23	1.5 (2)
C20—C8—C9—C1	−9.49 (18)	C24—C23—C28—C27	−0.1 (2)
C10—C1—C9—C5	130.18 (12)	N3—C23—C28—C27	176.03 (12)

### Hydrogen-bond geometry (Å, º)

**Table:**

*D*—H···*A*	*D*—H	H···*A*	*D*···*A*	*D*—H···*A*
O2—H2*A*···O1	0.87	2.14	2.8746 (14)	142
N3—H3···O3^i^	0.91	2.17	2.9362 (13)	141

Symmetry code: (i) *x*, −*y*+1/2, *z*−1/2.
